# Local delivery of insulin/IGF-1 for bone regeneration: carriers, strategies, and effects

**DOI:** 10.7150/ntno.46408

**Published:** 2020-09-08

**Authors:** Xiaoxuan Zhang, Helin Xing, Feng Qi, Hongchen Liu, Lizeng Gao, Xing Wang

**Affiliations:** 1 Shanxi Medical University School and Hospital of Stomatology, Taiyuan 030001, China.; 2 Shanxi Province Key Laboratory of Oral Diseases Prevention and New Materials.; 3 Department of Prosthodontics, Beijing Stomatological Hospital and School of Stomatology, Capital Medical University, Beijing, 100050, China.; 4 Department of Mechanical and Aerospace Engineering, University of Missouri, Columbia, MO, USA.; 5 Institute of Stomatology & Oral Maxilla Facial Key Laboratory, Chinese PLA General Hospital, 28 Fuxing Road, Beijing, 100853, China.; 6 CAS Engineering Laboratory for Nanozyme, Institute of Biophysics, Chinese Academy of Sciences, Beijing 100101, China.

**Keywords:** Nanoparticles, Insulin, Insulin-like growth factor, Bone regeneration, Angiogenesis, Osteogenesis

## Abstract

Bone defects caused by trauma, tumor resection, congenital malformation and infection are still a major challenge for clinicians. Biomimetic bone materials have attracted more and more attention in science and industry. Insulin and insulin-like growth factor-1 (IGF-1) have been increasingly recognized as an inducible factor for osteogenesis and angiogenesis. Spatiotemporal release of insulin may serve as the promising strategy. Considering the successful application of nanoparticles in drug loading, various insulin delivery systems have been developed, including (poly (lactic-co-glycolic acid), PLGA), hydroxyapatite (HA), gelatin, chitosan, alginate, and (γ-glutamic acid)/β-tricalcium phosphate, γ-PGA/β-TCP). Here, we have reviewed the progress on nanoparticles carrying insulin/IGF for bone regeneration. In addition, the key regulatory mechanism of insulin in bone regeneration is also summarized. The future application strategies and the challenges in bone regeneration are also discussed.

## Introduction

According to the world health organization, over 500,000 bone grafting procedures with a cost greater than $2.5 billion are performed annually in the United States 1. However, considerable clinical problems on bone defect are so far unaddressed, with a remaining group of 10% to 20% of delayed or nonunion situations 2. Owing to severe injury or congenital deformity, osteogenesis insufficiency, rheumatoid arthritis or osteoporosis, the long and limited self-healing process of bone grafts cannot fully meet the requirements of timely bone regeneration sometimes 3-5.

Insulin is a hormone that regulates energy balance and plays an important role in bone metabolism. Skeletal abnormalities associated with type I diabetes can be restored after insulin therapy 6, 7. Clinically, it is often observed that insulin deficiency is more likely to lead to fracture. In patients with type 2 diabetes mellitus, the use of insulin treatment significantly increased bone formation marker procollagen type 1 N-terminal propeptide, which can reduce the risk of fracture 8, 9. *In vivo* studies have shown that insulin/IGF-1 can promote angiogenesis and provide nutrition for bone formation 10-12. Insulin can effectively promote local skull bone formation by increasing the number of osteoblasts and osteoid surface area in the mouse skull 13, and can regulate the activity of osteoclasts 14. In recent years, studies have found that IGF-1 can also affect the growth and development of osteoblasts, accelerating bone regeneration 15.

**Figure 1:**
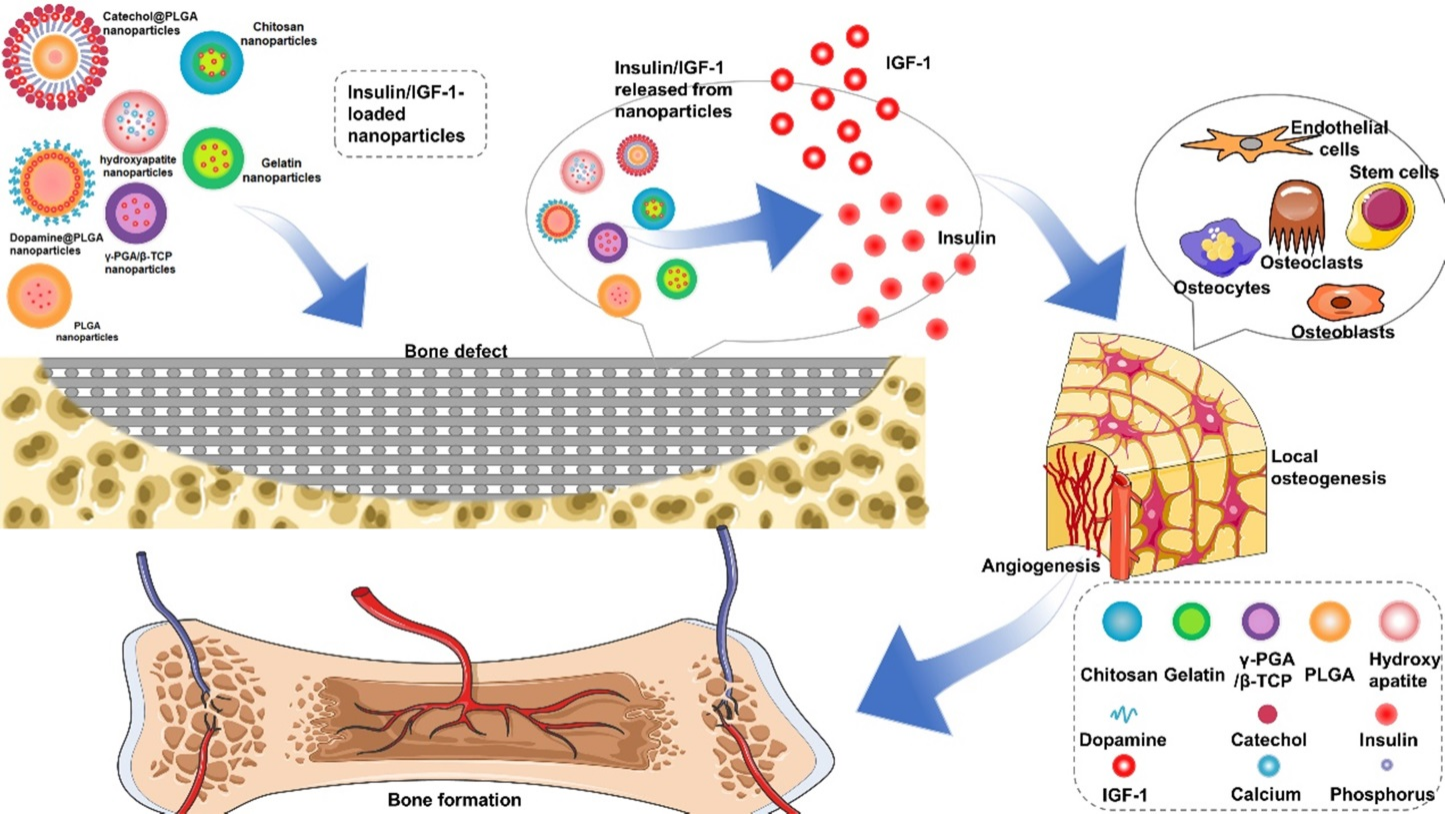
** Summarization of strategies for insulin delivery for bone regeneration.** Various nanoparticles-based delivery systems have been developed to improve the positive influence of insulin/IGF-1 on osteogenesis and angiogenesis.

However, the application of insulin in non-diabetic clinical bone defects has not received much attention, which may be due to the short life of insulin and the side effects of too much insulin. For example, the half-life of free insulin is only 20-30 min 16, 17. Encapsulated insulin in chitosan nanoparticles can enhance the absorption of insulin by stem cells and improve the hypoglycemic effect 18. This paper reviews the key regulatory mechanism of insulin in bone regeneration and the characteristics of different insulin loaded nanoparticles, such as: (poly (lactic-co-glycolic acid), PLGA), hydroxyapatite (HA), gelatin, chitosan, alginate, and (γ-glutamic acid)/β-tricalcium phosphate, γ-PGA/β-TCP) **(Figure 1)**. The application strategies in bone regeneration are emphasized and the challenges in the future are discussed.

## Potent role of insulin/IGF in bone regeneration

Insulin is derived from pancreatic beta cells, regulating mammalian cell growth and systemic energy metabolism 19. Insulin receptors (IR) are a transmembrane glycoprotein with tyrosine kinase activity. The binding of insulin with IR causes autophosphorylation of IR 20, 21, activating downstream phosphoinositide-3-kinase (PI3-K)/Akt and mitogen activated protein kinase (MAPK) pathways, which affect the differentiation, energy metabolism and activity of osteocytes 22, 23. Within a concentration range, insulin exposure could increase bone synthesis markers, including glucose uptake, alkaline phosphatase (ALP) production and collagen synthesis. Osteoblasts without IR have declined osteogenic differentiation, and reduced levels of alkaline phosphatase and runt-related transcription factor 2 (Runx2) 20. IGF is a group of growth-promoting polypeptide substances, whose secretory cells are widely distributed. IGF-1 has a molecular structure similar to insulin and possess cross-receptor to activate the downstream pathway, and IGF-1 can also bind IR and specifically activate insulin receptor substrate (IRS), a downstream insulin receptor substrate 24, 25.

### Insulin/IGF-1 promote osteogenesis

**Figure 2:**
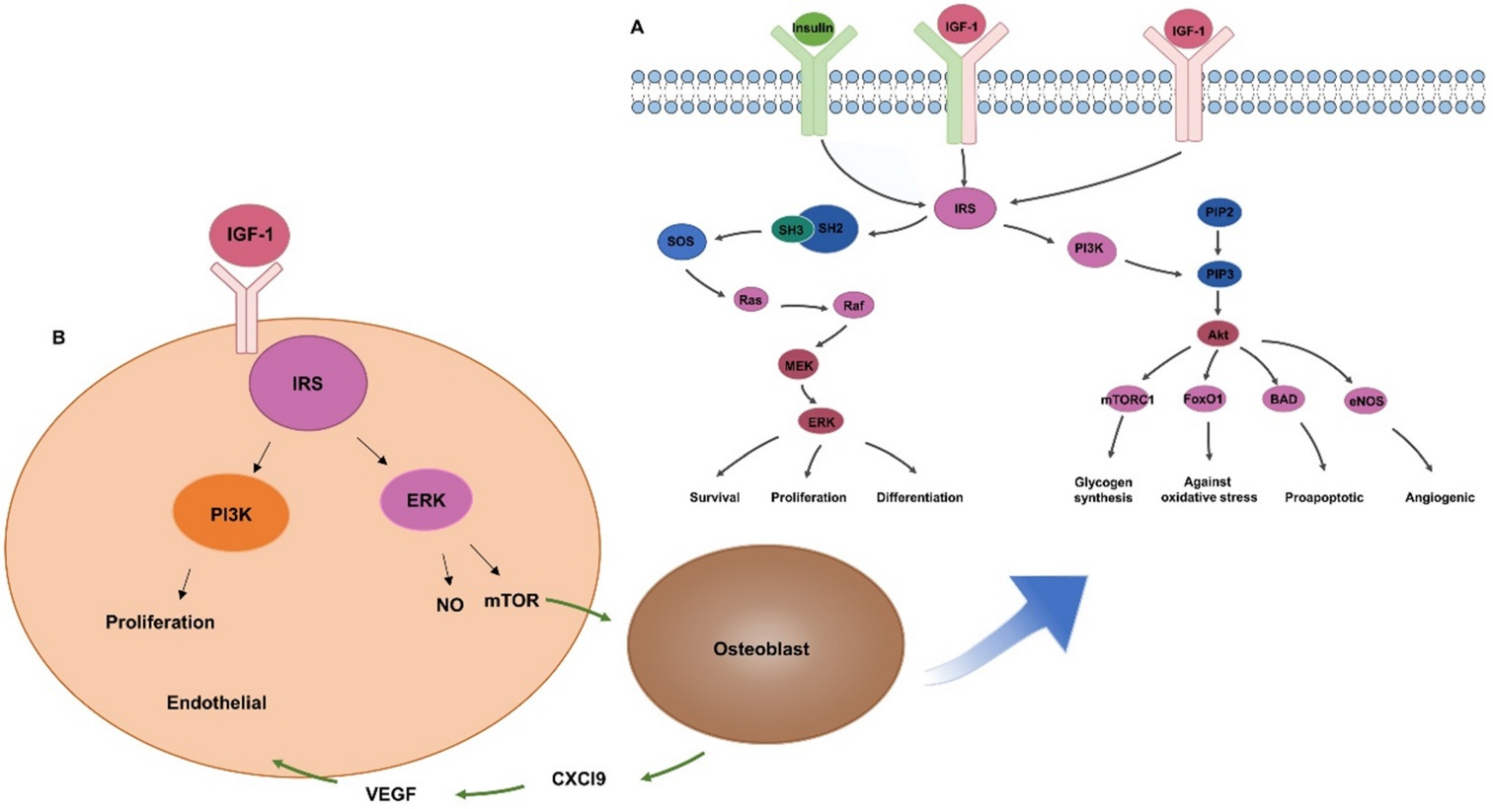
** Insulin / IGF-1 regulates bone regeneration through MAPK / PI3-K signaling pathway.** (**A**) Insulin / IGF-1 signal transduction in osteoblasts. (**B**) IGF-1 promotes angiogenesis by increasing endothelial cell proliferation.

Bone homeostasis is a dynamic balance that relies on tight interactions between osteoblasts and osteoclasts. IR exists in osteoblasts and osteoclasts, while IRS only exists in osteoblasts. IRS is a key factor in signal transduction of insulin and IGF-1 **(Figure 2A)**. It has a docking effect with Src homology 2 signal molecules in cells, transferring the signal through Src homology 3 to Son of Sevenless, phosphorylating downstream Ras molecules, thereby activating the extracellular signal-related kinases (ERK1/2) pathway and regulating the growth, proliferation, and differentiation of osteoblasts 26, 27. IRS tyrosine residues phosphorylation activates PI3-K 21, which means promotion of the transformation of phosphatidylinositol-4,5-bisphosphate to phosphatidylinositol-3,4,5-triphosphate. Phosphatidylinositol-3,4,5-triphosphate activates downstream AKT and then regulates the proliferation, survival and energy metabolism of osteoblasts through mammalian target of rapamycin, BCL-2-antagonist of cell death, forkhead box O1, and other pathways 28, 29. IRS in osteoblasts can regulate the generation of receptor activator of nuclear factor-kappa B ligand (RANKL), thereby regulating the differentiation of osteoclasts 23. Osteoblasts without insulin receptor substrate 1 cannot generate receptor activator of NF-kappaB ligand/osteoclast differentiation factor, which is critical for osteoclast development. The insulin receptor-deficient mice had been proved to show reduced bone resorption by osteoclasts, but the bone mass of these mice is still decreased, indicating that the inhibition of bone formation is stronger than that of bone resorption 19.

During bone regeneration, many transcripts must coordinate various factors to promote osteoblast differentiation, and Runx2 leads early stage differentiation 30. Runx2 induces osteoblast differentiation and enhances migration by interacting with PI3K-Akt signaling 31. Insulin signaling in osteoblasts plays a role by promoting the carboxylation of osteocalcin 19. Osteoblast insulin receptor (IR) signaling controls osteoblast development and osteocalcin expression by inhibiting the Runx2 inhibitor Twist2 20. Rapamycin complex 1 (mTORC1) is a highly conserved regulator of cell growth and is one of the most integrated signaling nodes in all cells. mTORC1 promotes cell growth by responding to nutrients and growth factors. Insulin has been found to activate mTORC1 through the PI3K-Akt pathway 32.

### Insulin/IGF-1 promote angiogenesis

In mammalian skeletal development, the formation of endochondral bone coincides with capillary invasion, suggesting a close relationship between osteogenesis and angiogenesis 33. In the osteogenic microenvironment, osteoblasts, osteoclasts, osteocytes and endothelial progenitor cells are involved in bone regeneration 34, 35.

Insulin and IGF-1 are important substance for promoting angiogenesis. Studies have shown that IR in endothelial cells promotes angiogenesis 36. Insulin-like growth factor receptors is also found in the endothelium 37 **(Figure 2B)**. IGF-1 binding to insulin-like growth factor receptors can activate downstream IRS and promote angiogenesis through the MAPK and PI3-K pathways 21, 38, 39. IGF-1 can activate ERK to promote the formation of vascular endothelial cells, and the release of nitric oxide by the PI-3K/Akt pathway to reduce vascular inflammation 40, 41. Chemokine (C-X-C motif) ligand 9 (Cxcl9) as a direct anti-regulatory molecule of VEGF signal generated by osteoblasts, regulating bone angiogenesis and osteogenesis. mTORC1 activates the expression of Cxcl9 by up-regulating STAT1 and increasing the binding of STAT1 to the Cxcl9 promoter in osteoblasts 42, suggesting that insulin might promote angiogenesis via Cxcl9.

In summary, vascularization and osteogenesis are important indicators of bone regeneration 43. Insulin/IGF-1 have potent roles in promoting bone regeneration, and therapeutic delivery of the factors should be beneficial.

## Application of insulin/IGF-loaded particles in bone regeneration

Nanoparticles is a promising strategy, which has been widely used to release drugs, maintain local concentration and improve bioavailability of insulin. Various nanoparticles loaded with insulin/IGF have been used for bone regeneration, such as those made of PLGA, HA, chitosan, gelatin, alginate, and γ-PGA/β-TCP nanoparticles. The preparation method, particle size, loading efficiency, encapsulation efficiency, and release behavior of the insulin-loaded nanoparticles are introduced in next part, as shown in **Table 1**.

### PLGA

In the 1970s, polymer particles were firstly applied involved in the field of biomedicine. Since 1997, the Food and Drug Administration and European medical institutions gradually approved the use of PLGA in drug delivery systems 44. The degradation rate of PLGA is mainly dependent on the ratio of glycolic acid to lactic acid monomer 45. To obtain more detailed information on accurate regulation of the release profile of insulin-loaded particles, three kinds of uniform-sized insulin microspheres were prepared using the solvent extraction, solvent evaporation and cosolvent methods, respectively 46. It was found that insulin-loaded microspheres prepared by the solvent evaporation method exhibited a relatively steady release rate during the first four weeks, which significantly stimulated the osteogenic differentiation of the stem cells and peri-implant bone regeneration.

**Figure 3:**
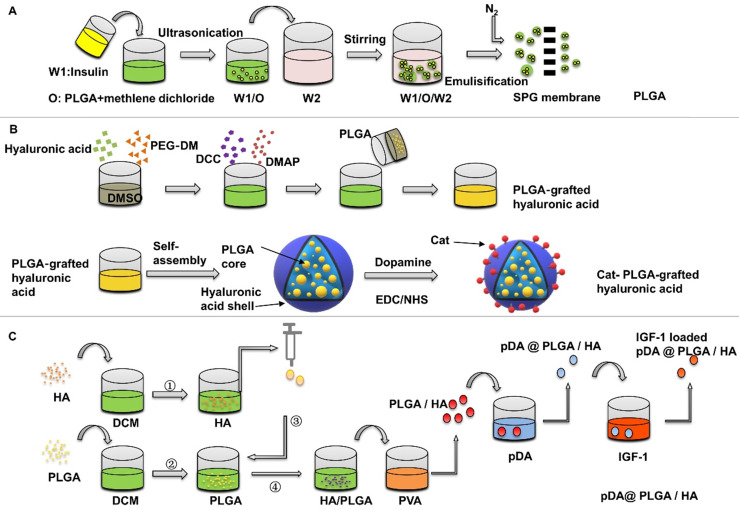
** Preparation of insulin-loaded PLGA nanoparticles.** (**A**) Insulin-loaded PLGA particles were prepared by Shirasu porous glass premixed membrane emulsification technology. Adapted with permission from 48, copyright 2017, American Chemical Society. (**B**) Preparation of Cat-HA-PLGA nanoparticles (**C**) pDA surface-modified PLGA/HA microspheres were prepared to deliver IGF-1.

Wang et al. 47 assessed the IGF-1 PLGA microspheres on bone healing around implants in type II diabetic rats. The preparation of PLGA is usually done by W/O/W double emulsion solvent evaporation process, which can be completed at room temperature. The average diameter of PLGA microspheres was 1.2525±0.6436 μm. The surface is smooth. The encapsulation efficiency was 78.3%. The initial burst release of the IGF-1 microspheres was 55.3% within 2 days, and more than 85% IGF-1 was released within 20 days. The efficacy of the drug-loaded microspheres was evaluated at 4 and 8 weeks, and the bone-implant contact percentage was found to be significantly increased in the microspheres group. In this study, the osteogenic effect was observed by suspending PLGA microspheres in the blood and then implanting the implants. The suspended PLGA microspheres may move before implanting the implants, resulting in uneven distribution. In addition, the size of nanoparticles also has a great influence on the release behaviour, but it has not been pointed out. Wang et al. 48 investigated the relationship between release kinetics of insulin and particle size of PLGA nanoparticles in bone regeneration. In this study, three kinds of insulin-loaded PLGA particles with narrow size distribution were prepared by Shirasu porous glass premixed membrane emulsification technology **(Figure 3A)**. The release kinetics showed that the initial release rate of PLGA microspheres with particle size of 1.61±0.08 μm was about 29% in the first day, and the remaining insulin ceased to release for 30 days, and accelerated bone defect healing *in vivo*, compared with those with sizes of 121.62 nm and 21.45 μm. Wang's experiment showed that PLGA has the best effect of releasing growth factor to promote osteogenesis at about 1 um, and in this study, A Shirasu Glass premixed membrane was specifically used to control the error to around 0.08, so that the particle size uniformity was improved and the release stability of the nano-microsphere was increased. Moreover, smaller particle size error can increase the repeatability of the experiment and has more potential in large-scale production.

#### Modification of PLGA

Although PLGA nanoparticles are commonly used in bone tissue engineering for drug delivery, the hydrophobic nature of PLGA is not conducive to cell adhesion, which needs further optimization 49. In order to optimize the effect of insulin-loaded PLGA on bone regeneration, many methods have been developed, including surface modification, and combination of PLGA to different materials 50.

Using surface modification is a common way to increase the protein adsorption of PLGA, Choi et al. 51 studied the PLGA-grafted hyaluronic acid nanoparticles with surface modified by catecholamine, which were synthesized by copolymerization method **(Figure 3B)**. The catechol groups on the surface of nanoparticles increase the adhesion on the surface of titanium. The negative-charged hyaluronic acid shell can absorb positive-charged IGF-1, which improves the loading capacity of IGF-1. The release rate of growth factor is related to the loading concentration. The initial burst release is 2% at 200 ng loading in the first 24 hours, and then reaches to 8% within 28 days slowly. In the same way, polydopamine (pDA) coating on the surface of particles can induce immobilization of some serum adhesives and adsorption of some cell types 52.

Recently, a multifunctional dopamine coating method has been developed, which only needs to immerse the substrate in alkaline dopamine solution 53. Catechol groups of dopamine are oxidized in an alkaline environment to form pDA layer on organic and inorganic materials, such as polymers, metal and metal oxides 54. pDA surface-modified PLGA/HA microcarriers with a diameter of 223.71±53.39 μm were prepared by Gao et al. 55, then adipose-derived mesenchymal stem cells were applied to detect the effect of pDA @ PLGA/HA/IGF-1 microcarriers on osteogenic differentiation **(Figure 3C)**. It was found that the proliferation and the expression levels of Runx2, osteopontin, and ALP of adipose-derived mesenchymal stem cells were significantly increased when cultured on functionalized PLGA/HA microspheres coated with pDA. The introduction of HA nanoparticles is of great significance for the design and preparation of biodegradable PLGA microcarriers. The addition of HA to PLGA could also improve the stability of PLGA degradation and provide a certain mechanical strength 56. Firstly, due to the improved surface roughness, addition of HA increases protein adsorption and cell proliferation. Secondly, surface pre-modification of microcarriers provides an optimization method for the immobilization of growth factor. Moreover, HA partially neutralized the local low pH value caused by degradation of PLGA microspheres, which reduced the effect on the structural integrity of the transferred IGF-1 57, 58. For PLGA microspheres, the selection of synthetic methods, particle size control and surface modification can increase the adsorption of insulin and IGF-1, optimize their release kinetics, and increase cell adhesion to achieve better osteogenic effect.

### HA

As the main component of natural bone, HA is widely used in orthopedics and dentistry to promote osteogenesis. The advantage of HA in bone is attributed to its structure and function similar to the mineral composition in bones and teeth. Due to its nano-structure characteristics and chemical affinity to molecules, ions and metal, HA is considered as a delivery system for peptides, proteins, antibiotics, drugs, and genes 59.

**Figure 4:**
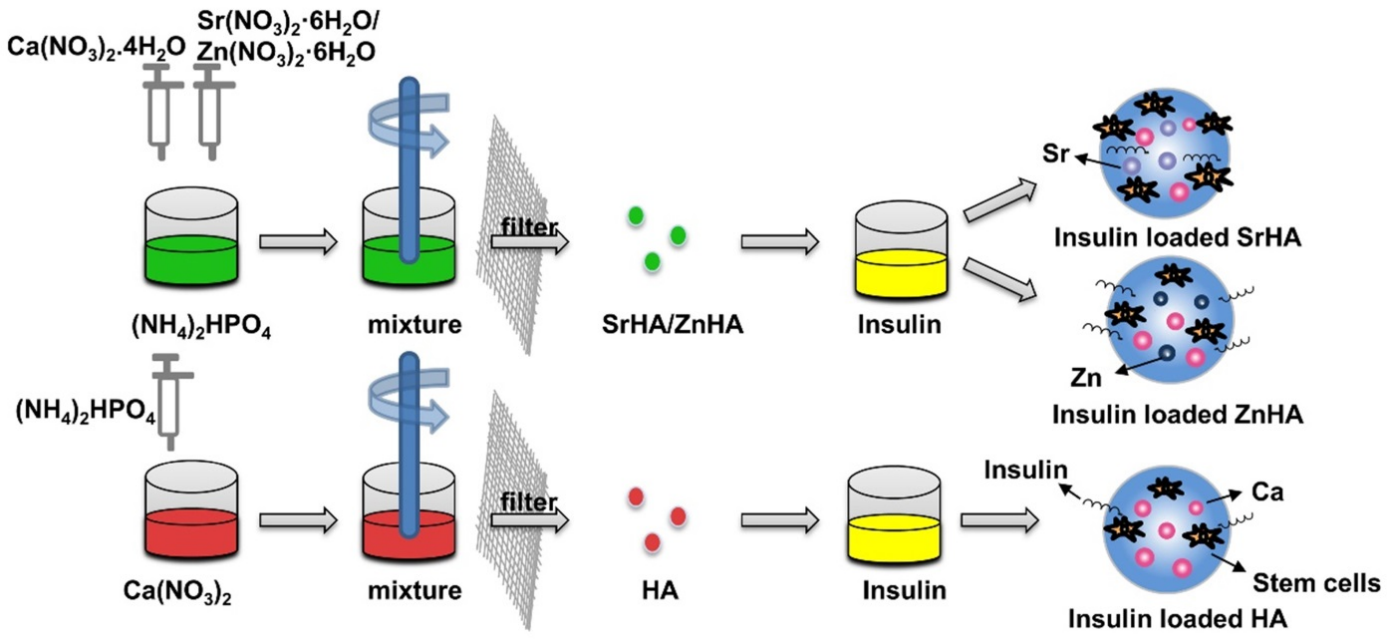
** Insulin delivery system based on HA nanoparticles.** Compared to the HA microspheres, the modification of zinc (ZnHA) enhance insulin adsorption capacity and strontium (SrHA) promotes stromal cell proliferation.

Insulin delivery system based on HA nanospheres has been widely used in blood glucose regulation 60-62. Some studies have found that adding insulin to HA can enhance the effect of HA on osteogenesis 63, 64. Lasgorceix et al. 65 studied the effects of HA and Si substituted HA granules loaded with insulin on osteoblasts, but no obvious cell proliferation was observed. Scudeller et al. 66 studied the adsorption effect of HA on insulin. In this study, strontium (SrHA) and zinc (ZnHA) were used on the surface to replace Ca^2+^ in HA. Results showed that the modification of zinc increased the surface area of HA microspheres and enhanced insulin adsorption capacity of HA microspheres. However, *in vitro* cell culture revealed that insulin-loaded SrHA promoted stromal cell proliferation, whereas insulin-loaded HA and ZnHA had no such effect, which was related to the fact that the substitution of strontium did not affect the structure of α helix of insulin **(Figure 4)**. In the delivery of insulin microspheres, HA is not easy to degrade, although have good mechanical strength, but not as good as gel polymer microspheres research widely, but in other studies, while testing different method for synthesis of microspheres osteogenesis effect, but no attention to the influence of insulin microspheres osteogenesis effect is due to the structure change of the insulin.

### Nanoparticles-hydrogel hybrid systems

Hydrogels have a wide range of applications from tissue engineering to drug delivery. Loading nanoparticles to produce composite hydrogels that can be used to customize and extend the mechanical properties of materials has been greatly developed. Nanoparticles have offered a unique set of properties for drug delivery including high drug loading capacity, combinatorial delivery, controlled and sustained drug release, prolonged stability and lifetime, and targeted delivery. To further enhance therapeutic index, especially for localized application, nanoparticles have been increasingly combined with hydrogels to form a hybrid biomaterial system for controlled drug delivery.

#### Gelatin

Gelatin is a commonly used natural polymer, which is derived from collagen and can be used in under different conditions of alkalinity, acidity and neutrality. It can be applied to transfer plasmid DNA, protein and other biological activity factors, and protect them from loss of bioactivity 67. Gelatin particles prepared by different methods have different degradation characteristics, which will affect the activity of the carried drugs. Moreover, gelatin easily dissolves rapidly in water, resulting in increased drug release. Nevertheless, use of a crosslinking agent is a major factor to maintain the stability of gelatin particles. Chemical crosslinking is a common solution, but the presence of residual crosslinkers may lead to toxic side effects 68.

**Figure 5:**
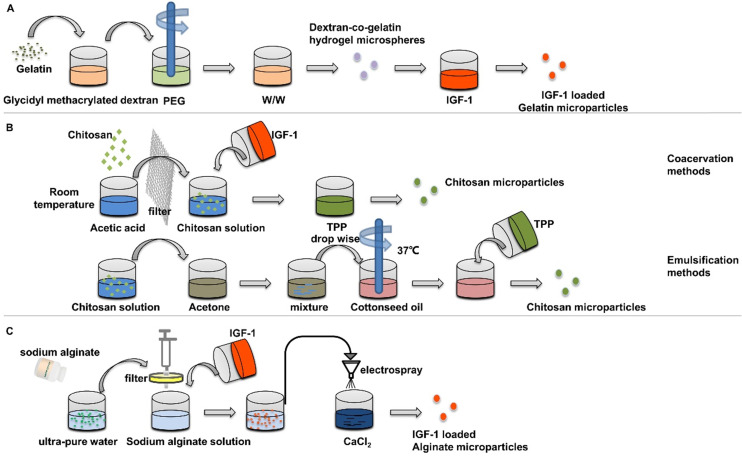
** Preparation of insulin-loaded nanoparticles using gelatin, chitosan and alginate.** (**A**) Gelatin Microspheres were prepared by an aqueous polyethylene glycol /dextran phase separation method. (**B**) Chitosan microparticles were prepared by emulsification and coacervation methods. (**C**) Insulin loaded alginate microparticles were prepared by electron spray method.

Using gelatin and dextran and glycidyl methacrylate, Chen et al. 69 mixed dextran-glycidyl methacrylate/gelatin solution with polyethylene glycol aqueous solution to form gelatin microspheres, then IGF-1 was adsorbed by swelling method **(Figure 5A)**. The particle size of the microspheres was mostly distributed in 30 μm. *In vivo* experiments of animals with periodontal defect, IGF-1 coated with gelatin particles was significantly more effective in bone formation than IGF-1 isolated in clot. The results showed that gelatin microspheres had a protective effect on IGF-1 and were used for drug delivery. Moreover, the use of dextran as a cross-linking agent can not only avoid the degradation of gelatin microspheres in IGF-1 solution, but also avoid the harmful effects caused by residues of chemical cross-linking agent.

To study cartilage repair by IGF-1-loaded nanoparticles, gelatin particles are also used to make hydrogel complexes of oligo(poly(ethylene glycol) fumarate) (OPF). Kim et al. 70 developed gelatin microparticles composite hydrogel particles by water oil method. After the adsorption of IGF-1, using glutaraldehyde crosslinking of macromolecule gel by double the hydrogel can be carried in cartilage repair two different kinds of growth factors, and the larger molecular weight reduce the density of gel, aperture, provide enough space for the cells to grow in, whether used alone or combined other growth factors, IGF-1 microspheres in the cartilage formation occupied the main role. The release of IGF-1 from OPF enhances the proliferation of MCF-7 cell lines and improves the growth of subchondral bone.

#### Chitosan

Chitosan is a kind of amino polysaccharide, which is obtained by alkaline N-deacetylation of natural macromolecule chitin 71. Chitosan and its derivatives have multiple advantages, such as biodegradability, non-toxic, good adhesion, renewable resources, low cost, which have been commonly used in food, medicine and pharmaceutics field, especially in the development of nano-drug delivery system.

IGF-1 has been proved to promote the biomineralization on the surface of chitosan nanoparticles 72, and the use of porous chitosan scaffolds to adsorb IGF-1 can effectively promote the healing of bone defects 73. Chitosan microspheres prepared under different conditions have different drug release rate. IGF-1-loaded chitosan microspheres can be prepared by emulsification and coacervation methods 74 **(Figure 5B)**. The amount of IGF-1 encapsulated by coagulated particles is about 50% more than that encapsulated by emulsified particles. After 14 days, only 3.31% of IGF-1 was released from the emulsified particles, while 30.68% from the coacervation particles, which indicated that organic solvents had adverse effects on the factors of the particles in the preparation process. In cell experiments, chitosan nanoparticles prepared by condensation method was cultured with osteoblasts. It was found that the nanoparticles enhanced the proliferation and differentiation of osteoblasts and increased the expression of osteogenic gene osterix.

When hydrogels with different components are used at the same time, a dual-transfer system with different factors can be obtained. Kim et al. 75 developed and characterized a chitosan gel/gelatin microspheres dual delivery system. The gelatin particles were prepared by water-in-oil emulsification, using glyoxal as a crosslinking agent to improve the stability of gelatin particles in a water environment. The obtained particles were encapsulated in chitosan solution to obtain chitosan/gelatin particles, and nanoparticles of 50 to 100 µm were further screened. The microspheres made of chitosan gel and gelatin can release bone morphogenetic protein-2 and IGF-1 in sequence, which can significantly improve the ALP activity of w-20-17 cells compared with that without microspheres. Synergistic growth factors can reduce the risk of overrelease of single factors and improve biosafety, but the optimal concentration and degradation properties of the factors carried by the dual delivery system have not been studied. Moreover, the above studies only demonstrated the promotion effect of osteogenesis mineralization at the cellular level, and the effect and toxicity level *in vivo* still need to be further evaluated.

#### Alginate

Alginate is a hydrophilic, biodegradable, natural polysaccharide with low toxicity that forms gel under gentle condition. Alginate possesses a variety of medical applications including cell encapsulation, drug stabilization and delivery 76, 77. Shilpi Goswami 78 studied the alginate loaded with insulin and directly mixed it with alginate solution using calcium chloride as cross-linking agent. The particle size of the nanoparticles obtained was not uniform, 40-150 nm. Moreover, it was found that when the amount of alginate added increased from 1 g to 2 g, hemolytic increased and biosecurity decreased. After loading insulin with the swelling method, the release was observed to be close to 60% within 30 min and 100% within 5 h. Duruel et al. 79 used electronic spraying method to prepare insulin-loaded alginate microparticles **(Figure 5C)**. The average diameter of the spherical alginate particles was 296±18 μm. The encapsulation efficiency was 40%, and the initial release rate was 60% in the first day. In this study, the longer release time was more appropriate for the slower cycle of osteogenesis. However, the author believes that in the sequential release, IGF-1 should be able to release continuously at the early stage, so alginate particles synthesized in calcium solution are a suitable choice. After ion exchange under other ions of microparticles in the medium, IGF-1 will be released by degradation of microspheres. Alginate particles and chitosan scaffolds were co-cultured with cementoblast cell line. The results showed that alginate particles containing IGF-1 promoted the expression of Runx2 compared with the control group containing chitosan alone.

#### γ-PGA/β-TCP

**Figure 6:**
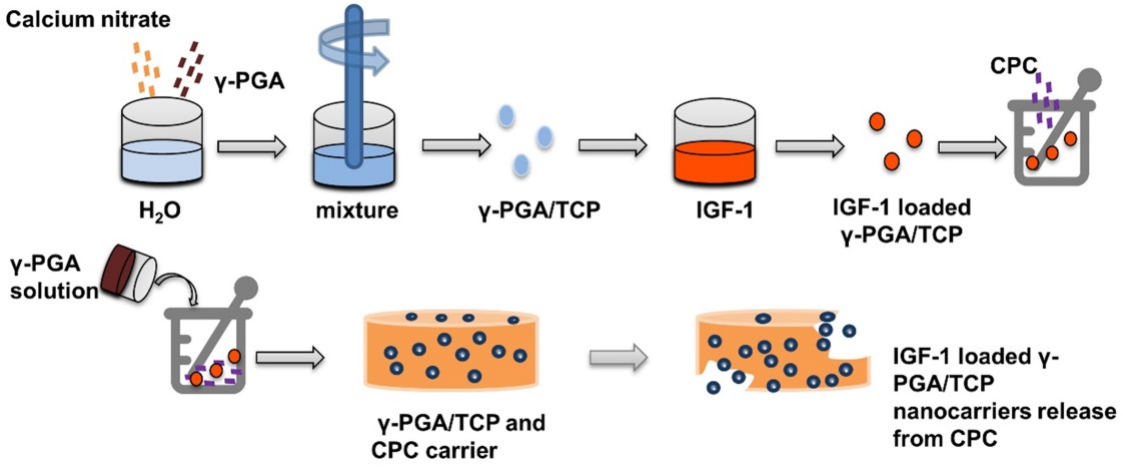
** Insulin delivery system based on γ-PGA / TCP nanoparticles:** Preparation and release of γ-PGA / TCP nanoparticles.

γ-glutamic acid (γ-PGA) has good expansibility and biocompatibility, and is suitable for clinical applications such as biogel, drug or gene delivery 80, 81. Lin et al. 82 used a simple and mildly ionic gelation method to make a kind of nano particle composed of γ-PGA and chitosan, which can effectively reduce blood glucose level by loading insulin. To moderate pore size, hardness, and flexibly, γ-PGA is combined with β-tricalcium phosphate to obtain γ-PGA/TCP nanoparticles for tissue engineering 83, 84. Shu et al. 85 prepared IGF-1 loaded γ-PGA/TCP particles, and then demonstrated that the nanoparticles increase the proliferation of MC3T3-E1 cells and ALP activity **(Figure 6)**.

Loading insulin into nanoparticles is a very important way to achieve desired insulin concentrations locally to improve the release time, but it is not enough to use nanoparticles alone in large area of bone defects, because it is difficult to maintain the morphology of defects. More research on osteogenesis is put into the combination of biological scaffolds and nano-microspheres. In the following section, we will highlight the strategy wherein nanoparticles are combined with scaffolds to improve the osteogenic effect and provide support for the defect.

## Scaffolds based insulin/IGF-1 delivery in bone regeneration

Bone grafts can be classified into allografts bone, autograft bone, and artificial bone. Autograft is a "gold standard" for clinical treatment due to its excellent osteogenesis, osteoinduction, and osteoconduction 86, but this method is limited by the amount of bone supplied at the site of extraction and causes additional damage to the bone area of the donor. Allogeneic bone graft can avoid secondary injury in the donor bone area, but it has the potential risk of infections by viruses 87. In recent years, artificial bone materials have become the focus of research on bone defect repair. Suitable artificial bone replacement materials should have the following conditions: (1) simulate the structure of natural bone, providing space for cell adhesion, angiogenesis and subsequent mineralization; (2) it has good supporting function; (3) appropriate degradation time to simulate the reconstruction of natural bone; (4) biocompatibility: immune behaviors such as rejection will not be induced; (5) promote osteogenic differentiation. Current studies include injectable hydrogel scaffolds, ceramic cement, polymer scaffolds, nanoparticle loaded scaffolds, etc.

HA is the component of bone, so its structure and hardness are the most similar to natural bone, but the brittleness of HA materials limits its application in bone tissue engineering 88, 89. And in the process of bone regeneration, it needs the continuous absorption of old tissue and the continuous mineralization of new tissue to carry out a functional reconstruction. Hydrogels are three-dimensional networks formed by hydrophilic homopolymers, copolymers or macromonomers. They swell in aqueous solution and provide an appropriate microenvironment similar to extracellular matrix, so as to promote the adhesion, migration, proliferation and differentiation of chondrocytes. Hydrogel can be implanted by minimally invasive injection and can form the desired shape to match irregular defects. Many natural materials such as chitosan, collagen or gelatin, alginate and hyaluronic acid can be used to prepare injectable hydrogels. Although hydrogels can adapt to different shapes of defects, their mechanical properties are relatively poor 90. They cannot provide necessary support when promoting bone formation. Therefore, the improvement of their mechanical properties needs further study. The synergistic effect of different components in nano doped biological scaffolds can solve the defects caused by single materials 89. Organo-silane and silk fibroin biopolymers can be assembled into multifunctional scaffolds. The incorporation of silk fibroin improves the porosity and mechanical elasticity of aerogel scaffolds, which can effectively support bone formation 91. However, it is not enough only to have good structural characteristics, but also to induce bone formation. There is a close relationship between nano materials and bone regeneration, especially in four main aspects: (1) Appropriate components in biomaterials can provide continuous nutrition for bone biomineralization 92. (2) Nano/microspheres can carry appropriate growth factors and promote bone mineralization 93. (3) Nano/microspheres combined with bone scaffolds can make up for the mechanical properties of bone scaffolds, such as adding PLGA microspheres to HA scaffolds 48. (4) Nano microspheres can release the growth factors gradually with the process of osteogenesis, which is more suitable for the process of osteogenesis 94.

In bone tissue engineering, artificial materials used to repair defects are often in the form of scaffolds, among which HA has mechanical properties similar to those of natural bone. Hydrogel scaffolds are biocompatible and more plastic, but the scaffolds alone can only guide bone rather than induce bone regeneration. Insulin-loaded nanoparticles can improve the mechanical properties of bone scaffolds and release growth factors in a controlled manner, making up for the shortcomings of osteogenic scaffolds. Thus, a composite scaffold of insulin-loaded particles and porous scaffold may be a preferable strategy **(Table 2)**.

**Figure 7:**
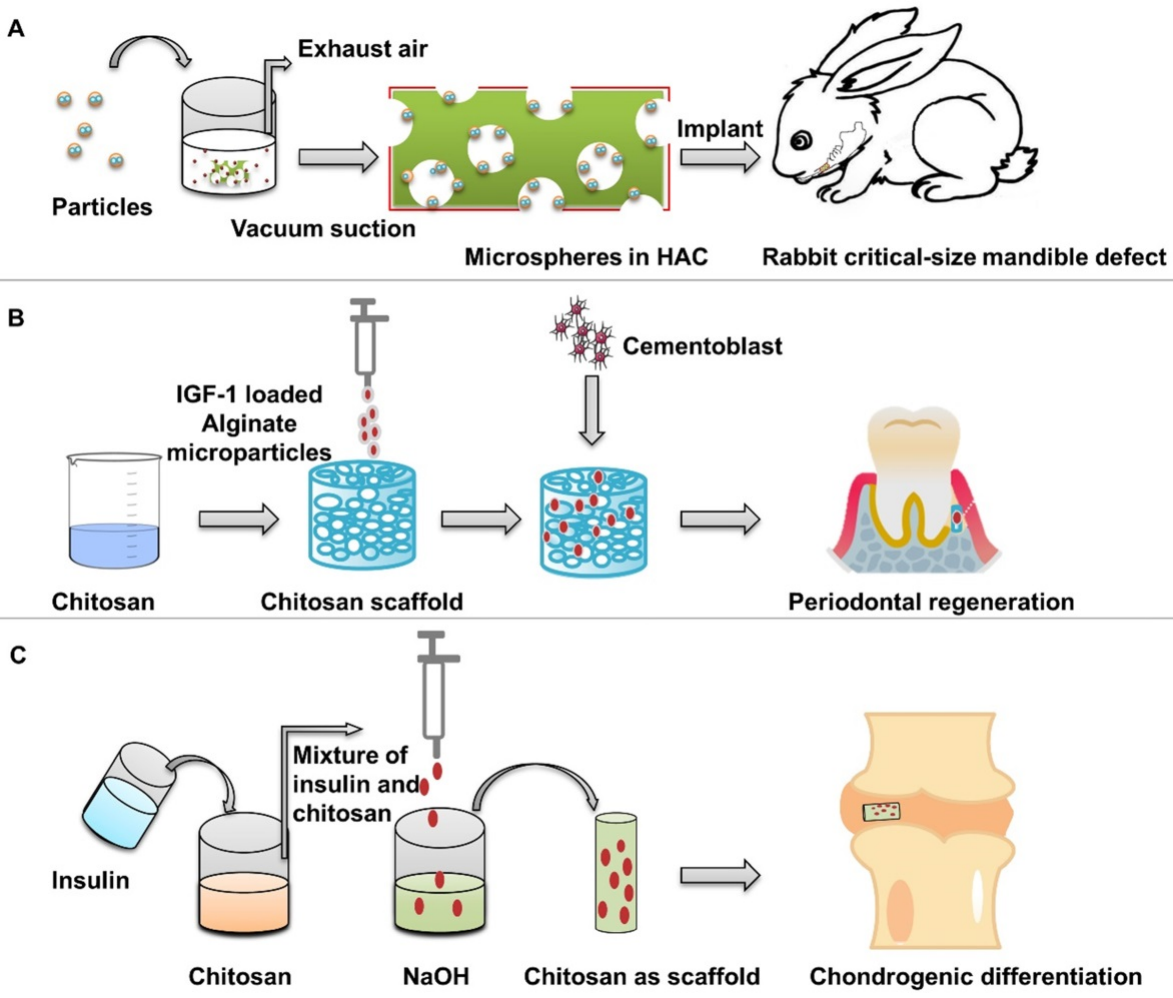
** Preparation of insulin-loaded composite scaffold.** (**A**) Composite scaffold was prepared using insulin-loaded PLGA microspheres and nHAC scaffolds. Adapted with permission from 48, copyright 2017, American Chemical Society. (**B**) Composite scaffold was prepared using IGF-1 loaded alginate microspheres and chitosan scaffolds. (**C**) Preparation of insulin-loaded chitosan scaffold.

Wang et al. 48 used the nano-hydroxyapatite/collagen (nHAC) as the scaffold of insulin-loaded PLGA particles to repair the critical defect of rabbit mandible **(Figure 7A)**. At 8 weeks, the new bone filled and mineralized the defect. nHAC/PLGA composite scaffolds have good mechanical and structural characteristics in the repair of large jaw defects, which can maintain the morphology of the defect site, promote cell adhesion on the scaffolds, and release insulin at a stable rate to promote the differentiation of osteoblasts.

The prepared nano-microspheres were placed in a polymer solution, and then the mixture was placed in the mold and dried to form a scaffold with a specific shape to carry the microspheres. As a carrier of growth factor, gelatin particles can be deposited in the hydrogel to form a solid with fixed shape, which can provide the release of IGF-1 under the action of collagenase 95. The scaffolds synthesized by this method do not require high temperatures 96, but the crosslinking agents may be harmful to organisms. Chen et al. 97 used IGF-1 loaded dextran-glycidyl methacrylate/gelatin composite hydrogel scaffolds instead of single microspheres to obtain more non-toxic composite scaffolds. Suspended particulate matter in OPF can be used to obtain integral scaffolds by crosslinking agent without high temperature, and avoid the decrease in activity caused by thermal denaturation of bioactive factors 70. This method has short crosslinking time, and is easy to obtain bilayer scaffolds.

Duruel et al. 79 prepared chitosan scaffolds by freeze-drying method. The internal connection porosity of the scaffold was 80%. Sodium alginate particles loaded with IGF-1 were integrated into the scaffold structure, which induced the proliferation and differentiation of osteoblasts** (Figure 7B)**. Malafaya et al. 98 used insulin-loaded chitosan particles to directly form scaffolds by drying method. Insulin solution and chitosan polymer were mixed, and then dripped into NaOH by a syringe to obtain insulin-loaded chitosan particles **(Figure 7C)**. Then the chitosan particles were placed in the mold and dried at 60 °C to obtain a scaffold with a specific morphology formed by the materials of the particles. Chitosan scaffolds release insulin in a dose-dependent manner to support the adhesion and differentiation of chondrocytes and the biosynthesis of cartilage matrix. The encapsulation efficiency of insulin was 87.23%. When the insulin loadings were 5%, the release of chitosan particles was relatively stable.

nHAC scaffolds have high strength and are suitable for the repair of hard bone defects. The hydrogel was mixed with nanoparticles, the scaffolds with a more uniform distribution of nanoparticles were obtained by direct solidification. The degradation of hydrogel scaffolds is more suitable for the simultaneous existence of cartilage defects. Moreover, it is easy to get the desired shape and can obtain double-layer scaffolds with different growth factors.

## Conclusion and perspectives

**Figure 8:**
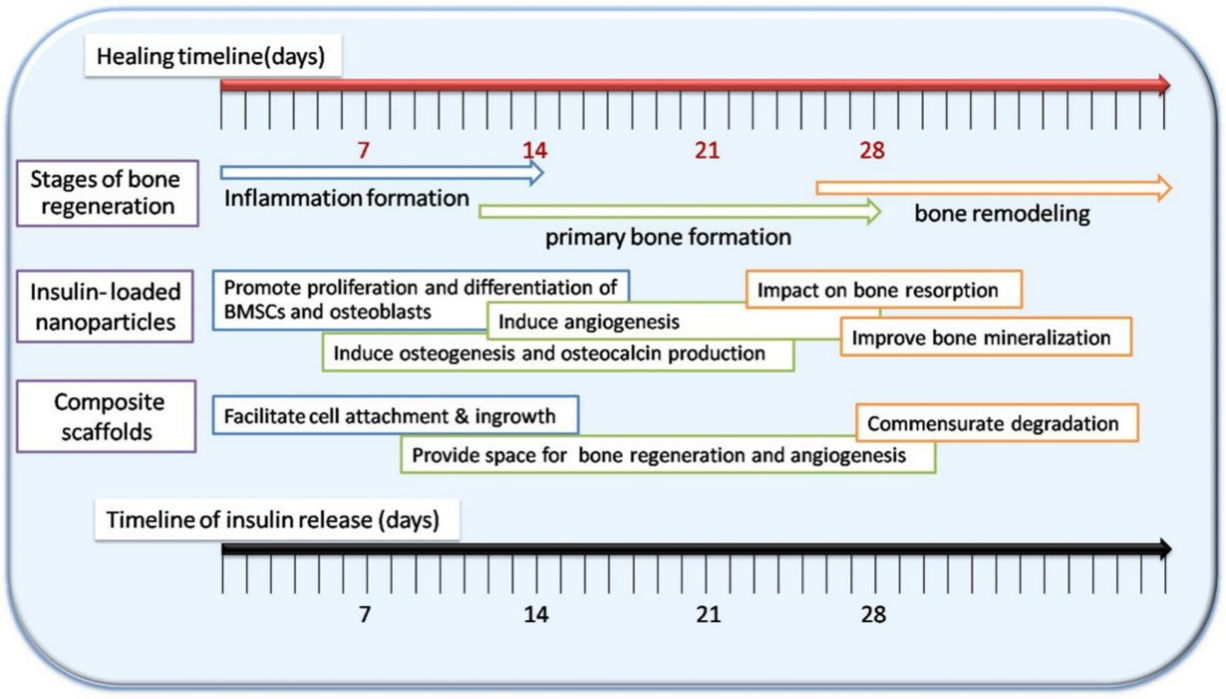
** Phases of bone regeneration and the capacity of insulin-loaded nHAC/PLGA composite scaffolds.** Adapted with permission from 48, copyright 2017, American Chemical Society.

To summarize, insulin and IGF-1 have been increasingly recognized as an inducible factor for osteogenesis and angiogenesis, which is expected to be a preferable drug for bone regeneration. Nanoparticles have been widely studied in sustained release, which can provide opportunities for regulating local insulin concentration and have great potential in bone regeneration **(Figure 8)**. This review presents the latest advances of insulin-loaded nanoparticles, including PLGA, HA, gelatin, chitosan, alginate, and γ-PGA/β-TCP. Information on nanocarriers introduced in this review is summarized in **Table 1**. Information on scaffold introduced in this review is summarized in **Table 2**. It is important to note that there remain challenges to be fulfilled. For example, the loading efficiency remains to be improved. In addition, the use of insulin-loaded nanoparticles alone is difficult to provide support and repair of large-scale defects. Strategies to combine insulin carrying nanoparticles and scaffolds are needed. In order to control insulin release at the optimal time, a lot of improvements are undergoing.

In conclusion, bone regeneration is a long-term and complex process, and local delivery of insulin with various strategies would certainly promote the process, and produce beneficial effects in bone regeneration.

## Figures and Tables

**Table 1:** Characteristics of insulin/IGF-1 nanoparticles

Carrier	Synthesis Method	Components	Drug	LE/EE	Size	Release behaviour* in vitro*	Effect in bone regeneration	Ref
PLGA	W/O/W system	DCM + PVA + PLGA	IGF-1	EE = 78.3%	**-**	Initial burst release (55.3%, 48 h); More than 85% was released within 20 days.	Promote osseointegration of dental implants.	47
PLGA	(W/O/W) system with SPG premix membrane	DCM + PVA + PLGA	Insulin	LE = 5.4% EE = 60.2%	121.2 nm	Initial burst release (51%, 24 h) followed drug release over 5 days.	Bone mineral density (8 week): 0.96 ± 0.06 g/cm^3^.	48
				LE = 28.27% EE = 67.86%	1.61 μm	Initial burst release (29%, 24 h); Sustained release over 30 days.	Bone mineral density (8 week): 1.10 ± 0.07 g/cm^3^.	
				LE = 33.72% EE = 93.29%	21.45 μm	Initial lower burst (18%, 24 h); within a long period up to 2 weeks, the release rate was slow, but then increased in the second month.	Bone mineral density (8 week): 0.86 ± 0.07 g/cm^3^.	
HA	Dropwise aqueous	Ca(NO_3_)_2_/Sr(NO_3_)_2_/Zn(NO_3_)_2_	Insulin	**-**	< 210 μm	**-**	SrHA/insulin promoted stem cell proliferation.	66
PLGA /HA	Copolymerization method	HA + PEG-DM + DCC + DMAP	IGF-1	LE = 99%	131.3 nm	Initial burst release (4g within 24 h); Sustained release within 28 days.	Increase proliferation of ADSCs on Ti substrates.	51
PLGA /HA	O/W emulsion + pDA coating	HA + DCM + PLGA + PVA + DA	IGF-1	**-**	223.71 μm	**-**	Increase proliferation, adhesion, and osteogenic differentiation of ADSCs.	55
Gelatin	W/W emulsion	PEG + Dex-GMA/gelatin	IGF-1	LE = 5%	30 μm	Initial burst release: 20.4 ± 1%, 24 h release: > 28 days.	Enhance periodontal tissue regeneration.	69
Gelatin	Swell + crosslink	OPF + Gelatin microparticles	IGF-1		50-100 μm	Initial burst release (30%, 24 h); 1% each day: (days 18-28).	Improves the subchondral bone morphology and the interaction with the surrounding chondral tissue.	70
Gelatin	Heating crosslink	OPF + Gelatin + Glutaraldehyde	IGF-1	Loading 100-200 ng: EE = 100%	50-100 μm	Initial burst release: (22%, 24 h); Cumulative release 28 day.	**-**	95
Chitosan	Emulsification method	Chitosan + Cottonseed oil + TPP	IGF-1	70.02 ng	50-80 μm	3.31% (14 d)	**-**	74
	Coacervation method	Acetic acid + Chitosan + TPP	IGF-1	168.7 ng	500-700 μm	30.68% (14 d)	Enhance pre-osteoblasts proliferation/differentiation.	
Chitosan	W/O emulsion	Gelatin + Glyoxal + Chitosan	IGF-1	**-**	**-**	Initial burst release: 48%, 24 h; Sustained release 7 days.	Increase ALP activity of W-20-17 cells.	75
Alginate	Electrospraying method	Sodium alginate chitosan flakes	IGF-1	EE = 40%	296 ± 18 μm	60% within 24 h	Promote the proliferation and differentiation of cementoblasts.	79
**γ**-PGA/β-TCP	Two-step desolvation	γ-PGA + β-TCP	IGF-1	**-**	**-**	52.86% (12 d)	Sequential release of BMP-2 and IGF-1 significantly promoted the proliferation and differentiation of MC3T3-E1 cells.	85

ADSCs: adipose-derived mesenchymal stem cells; ALP: alkaline phosphatase; BMP-2: bone morphogenetic protein 2; DA: dopamine; DCM: dichloromethane; DCC: 1,3-dicyclohexylcarbodiimide; Dex-GMA: dextran-glycidyl methacrylate; DMAP: 4-dimethylaminopyridine; HA: hydroxyapatite; IGF-1: insulin-like growth factor 1; LE/EE: loading efficiency/encapsulation efficiency; OPF: oligo(poly(ethylene glycol) fumarate); pDA: polydopamine; PEG: polyethylene glycol; PEG-DM: Poly(ethylene glycol) dimethyl ether; PLGA: poly (lactic-co-glycolic acid); PVA: polyvinyl alcohol; **γ**-PGA/β-TCP: (γ-glutamic acid)/β-tricalcium phosphate; SPG: Shirasu porous glass; SrHA: strontium substitution in HA; TPP: tripolyphosphate; W/O/W: water/oil/water.

**Table 2:** Insulin/IGF-1 composited scaffolds in bone regeneration

Nanoparticles	Scaffold	Drug	Characterizations of scaffold	Release behaviour of scaffold	Effect in bone regeneration	Ref
PLGA	nHAC/PLGA	Insulin	Nano-nHAC pore size: 127.4 ± 21.1	Initial burst release (37% within 24 h); Constant release within 10 days.	Enhance proliferation and osteogenesis of BMSCs *in vitro*; Restore critical Size bone defect *in vivo*.	48
		Insulin	Micron-nHAC pore size: 122.2 ± 11.4 μm	Initial burst release (23% within 24 h); Sustained release over the next 30 days.		
		Insulin	10×Micron-nHAC pore size: 97.2 ± 20.9 μm	Initial burst release (16% within 24 h); Sustained release over the next 50 days.		
Gelatin	Dex-GMA/Gelatin	IGF-1	Loading 5 ng IGF-1 per mg of freeze-dried scaffold	Sustained release during 28 days.	Promoting the proliferation, metabolism and ALP activity of periodic metabolism fibroblasts.	97
Gelatin	OPF	IGF-1	Degradation: remained stable over 28 days in PBS; started to increase after 7 days in collagenase-PBS	Initial released (36.6 ± 8.8%, 4 days) Sustained release during 28 days.	Enhance cartilage morphology in an osteochondral defect.	70
Chitosan	Chitosan	Insulin	Scaffolds with 3-mm-height and 5-mm-diameter cylindrical shape	5% insulin initial released: (40%, the first 4 days); 85% insulin was sustained at 28 days.	Enhance cartilage morphology and the cartilaginous genes Sox-9.	98
Alginate/ PLGA	Chitosan	IGF-1	Pore size:30-150 μm Porosity of the scaffold: 80%	**-**	Increased ECM synthesis, cell differentiation and mineralize nodule formation.	79

ALP: alkaline phosphatase; BMSCs: bone marrow derived mesenchymal stromal cells; Dex-GMA: dextran-glycidyl methacrylate; ECM: extracellular matrix; IGF-1: insulin-like growth factor 1; nHAC: nano-hydroxyapatite/collagen; OPF: oligo(poly(ethylene glycol) fumarate); PBS: phosphate buffered saline; PLGA: poly (lactic-co-glycolic acid).

## Abbreviations

ALP: alkaline phosphatase
Cxcl9: chemokine (C-X-C motif) ligand 9
ERK1/2: extracellular signal-related kinases
HA: hydroxyapatite
IGF: insulin and insulin-like growth factor
IGF-1: Insulin-like growth factor 1
IGFBPs: insulin like growth factor binding proteins
IR: insulin receptors
IRS: insulin receptor substrate
MAPK: mitogen activated protein kinase
mTORC1: rapamycin complex 1
nHAC: nano-hydroxyapatite/collagen
OPF: oligo(poly(ethylene glycol) fumarate)
pDA: polydopamine
PI3-K: phosphoinositide-3-kinase
PLGA: poly (lactic-co-glycolic acid)
RANKL: activator of nuclear factor kappa B
γ-PGA/β-TCP: (γ-glutamic acid)/β-tricalcium phosphate
Runx2: runt-related transcription factor 2
SrHA: strontium substitution in hydroxyapatite
ZnHA: zinc substitution in hydroxyapatite
